# Physician Perceptions Toward Palliative Care Integration in Childhood Cancer Care in Asia Pacific

**DOI:** 10.1200/GO-25-00513

**Published:** 2026-06-26

**Authors:** Marta Salek, Andrea Cuviello, Sri Andini Handayani, Sally Blair, Anjali Chandra, Godwin Job, Bella S. Ehrlich, Lee Ai Chong, Poonam Bagai, Ong Gek Bee, Shella Bravo, Siti Nur Hanim Buang, Huyen Thi Thanh Bui, Lynna Chandra, Tashi Choden, Ross Drake, Bishnu Rath Giri, Dylan E. Graetz, Sanjeeva Gunasekera, Haresh Gupta, Chong Poh Heng, Mariko Kakazu, Sung Han Kang, Erica C. Kaye, Chusana Khaiman, Aye Aye Khaing, Min Sun Kim, Nia Kurniati, Stephen Laughton, Lurdes Maria do R. Leão, Amita Mahajan, Michelle Cristine Miranda, Ratha Mlis, Thida Moe, Mimi Lhamu Mynak, Lan Bui Ngoc, Hoa Thi Kim Nguyen, Ximena Garcia Quintero, Eman Rasheed, Diana Rios, Milena Maria Lay Dos Santos, Sudhir Sapkota, Teny Tjitra Sari, Krishna Sagar Sharma, Sani Sipai, Rojim Sorrosa, Bounpalisone Souvalansy, Pornpun Sripornsawan, Sommaphun Tabjaroen, Teresa Shu Zhen Tan, Kok Hoi Teh, Rina Wahyuni, Kazuyo Watanabe, Su Yadana, Wynn Yi Yi, Nobuyuki Yotani, Maziya Abbas Zaki, Meenakshi Devidas, Justin N. Baker, Michael J. McNeil, Asya Agulnik, Catherine G. Lam

**Affiliations:** ^1^ St Jude Children’s Research Hospital, Memphis, TN; ^2^ Phoenix Children’s Hospital, Phoenix, AZ; ^3^ Stanford University School of Medicine, Palo Alto, CA; ^4^ Boston Children’s Hospital, Boston, MA; ^5^ Universiti Malaya, Kuala Lumpur, Malaysia; ^6^ CanKids KidsCan, Delhi, India; ^7^ Sabah Women and Children’s Hospital, Sabah, Malaysia; ^8^ Southern Philippines Medical Center, Davao City, Philippines; ^9^ KK Women's and Children's Hospital, Singapore, Singapore; ^10^ VinUniversity, Hanoi, Vietnam; ^11^ Rachel House Foundation (Yayasan Rumah Rachel), Jakarta, Indonesia; ^12^ Jigme Dorji Wangchuck National Referral Hospital, Thimphu, Bhutan; ^13^ Queensland Children’s Hospital, Brisbane, Australia; ^14^ World Health Organization Regional Office for Southeast Asia, New Delhi, India; ^15^ National Cancer Institute, Maharagama, Sri Lanka; ^16^ HCA Hospice Care, Singapore, Singapore; ^17^ Japan Heart Children’s Medical Center, Kandal, Cambodia; ^18^ Asan Medical Center Children’s Hospital, University of Ulsan College of Medicine, Seoul, Republic of Korea; ^19^ Chulalongkorn University, Bangkok, Thailand; ^20^ Yangon Children's Hospital, Yangon, Myanmar; ^21^ Seoul National University Hospital, Seoul, South Korea; ^22^ Dr Cipto Mangunkusumo Hospital, Jakarta, Indonesia; ^23^ Starship Blood and Cancer Center, Auckland, New Zealand; ^24^ National Hospital Guido Valadares, Dili, Timor-Leste; ^25^ Philippine General Hospital, Manila, Philippines; ^26^ Calmette Hospital, Phnom Penh, Cambodia; ^27^ Vietnam National Children's Hospital, Hanoi, Vietnam; ^28^ Hu General Hospital, Hue City, Vietnam; ^29^ Hulhumale’ Hospital, Hulhumalé, Maldives; ^30^ Kanti Children’s Hospital, Kathmandu, Nepal; ^31^ B.P. Koirala Memorial Cancer Hospital, Bharatpur, Nepal; ^32^ Children's Hospital, Vientiane, Laos; ^33^ Prince of Songkla University, Hat Yai, Thailand; ^34^ Thai Pediatric Oncology Group, Bangkok, Thailand; ^35^ Khoo Teck Puat-National University Children's Medical Institute, National University Hospital, Singapore, Singapore; ^36^ Hospital Tunku Azizah, Kuala Lampur, Malaysia; ^37^ Asian Children's Care League, Tokyo, Japan; ^38^ National Center for Child Health and Development, Tokyo, Japan

## Abstract

**PURPOSE:**

Palliative care (PC) integration in pediatric oncology improves outcomes, yet access to PC in the Asia Pacific (AP) region remains limited. This study aimed to understand physician perceptions of PC integration in childhood cancer care across AP.

**METHODS:**

The validated Assessing Doctors' Attitudes on Palliative Treatment survey was modified for use in AP, translated into six languages, and adapted for cultural relevance. The survey was distributed electronically between February 2022 and February 2024 to physicians caring for children with cancer in 18 AP countries. The primary outcome was alignment with WHO PC guidance, calculated as a mean percentage per physician. Secondary analyses explored associations between demographic variables and WHO alignment scores using regression analyses. Open-ended responses were analyzed qualitatively.

**RESULTS:**

A total of 621 physicians from 18 countries participated (median country response rate 30%; range, 11%-85% per country). Most (70%; n = 432) had >10 years of clinical experience and no prior PC training (65%; n = 401). Although 57% (n = 352) had access to pediatric PC experts, only 50% (n = 308), 36% (n = 221), 27% (n = 166), and 34% (n = 209) expressed comfort addressing patient/family physical, emotional, spiritual, and grief/bereavement needs, respectively. Additionally, 40% (n = 248) reported feeling burdened addressing end-of-life suffering. The mean alignment with WHO guidance was 76% (range, 48%-92%). Almost all (>90%; n = 570) desired further PC training.

**CONCLUSION:**

Study findings demonstrate that most physicians in AP expressed discomfort with providing core components of PC to patients with cancer and their families. This study signals the urgent need for focused expansion of PC services and training and identifies regional opportunities to improve PC education, research, and advocacy efforts.

## INTRODUCTION

Palliative care (PC) is an essential component of high-quality care for children facing serious illness and their families.^1,2^ PC aims to enhance quality of life by preventing and relieving physical, psychosocial, and spiritual suffering and is recognized as an element of the fundamental human right to health.^3-8^ Despite the adoption of multiple international resolutions and political declarations on PC by member states over the past decades—including those from the WHO and United Nations—progress in expanding PC access remains limited, especially in resource-constrained settings.^3-11^ Globally, there are an estimated 20 million children in need of PC. Approximately 30% of these children live in the Asia Pacific (AP) region.^12^

**CONTEXT**

**Key Objective**
This mixed-methods study aimed to explore physicians' perspectives on integrating palliative care (PC) into childhood cancer treatment across the Asia Pacific (AP) region.
**Knowledge Generated**
A total of 621 physicians from 18 AP countries participated. Although most physicians could identify core PC components, some held misconceptions and were unsure when to involve PC teams. Many felt uncomfortable addressing patients' and families' PC needs, but those with prior PC training were more confident and resilient. Nearly all expressed a strong desire for additional PC education.
**Relevance**
These findings underscore the need for country- and region-specific strategies in AP to strengthen PC capacity, inform policy and research, and guide the development of tailored training programs for clinicians caring for children with cancer. Applying these insights in the clinical context can enhance multidisciplinary care, improve patient and family outcomes, and support clinicians in delivering comprehensive, compassionate care across the disease trajectory.

Children diagnosed with cancer contribute to the population in need of PC. Across AP, approximately 72,000 children are diagnosed with cancer annually and many of these lack access to PC services.^9,12,13^ AP is a large, socioculturally diverse region spanning two WHO regions of South-East Asia and Western Pacific with differences in models of health care delivery and available resources. Differences in health care delivery are reflected in diverse approaches to interdisciplinary, PC provision reflecting variation in patient, family, and community needs and preferences.^14^ Physician perceptions of PC integration into childhood cancer care within the region have not been previously explored. Understanding physician perceptions toward PC integration is crucial to improve PC access and ultimately patient outcomes.

The Assessing Doctors' Attitudes on Palliative Treatment (ADAPT) mixed-methods survey explores physician perceptions toward PC integration in childhood cancer. This survey was initially developed and implemented in Eastern Europe and Central Asia, where study findings led to numerous education and advocacy initiatives that addressed PC misconceptions and barriers to care provision regionally.^15-18^ As a result, the survey has been adapted to other world regions, including Latin America and Western Europe.^19-22^ Similarly, the aim of this mixed-methods study was to explore physician perspectives and comfort with integrating PC into childhood cancer care across AP using the previously validated ADAPT survey.

## METHODS

The research team included the coordinating core research team, nine-member expert panel, and country leaders, comprising 45 pediatric oncology, PC, and foundation experts from 18 countries across AP (Bhutan, Cambodia, India, Indonesia, Japan, Laos, Malaysia, Maldives, Myanmar, Nepal, New Zealand, Philippines, Republic of Korea, Singapore, Sri Lanka, Thailand, Timor-Leste, and Vietnam). The expert panel and country leaders were identified through the St Jude Global Alliance Community, an international network of over 400 medical institutions and foundations from more than 80 countries dedicated to improving outcomes for children facing catastrophic illness globally.^23^ The expert panel supported adaptation of the survey instrument for distribution in AP. Each participating country had a team of one to four multidisciplinary specialists who supported study planning, survey dissemination, participant recruitment and communication, and study logistics coordination.

This study was deemed exempt by the Institutional Review Board at St Jude Children's Research Hospital in Memphis, Tennessee. During study planning, participating institutions in 12 countries, including India, Indonesia, Japan, Laos, Malaysia, Myanmar, Nepal, New Zealand, Philippines, Sri Lanka, Timor-Leste, and Vietnam, identified the need to obtain local ethical approvals. The study adhered to the American Association for Public Opinion Research reporting guidelines.^24^

### Instrument Development and WHO Alignment

The ADAPT study uses a mixed-methods tool that collects quantitative and qualitative data in a convergent parallel design. The survey was originally created by an international panel of pediatric oncology and PC experts to assess physicians' perceptions of PC provision in childhood cancer care in Eastern Europe and Central Asia, with study findings published in 2020.^15^ Survey items were based on existing WHO guidelines for PC integration in health care and a literature review exploring physician perceptions of PC integration.^15^ This survey has been distributed in Eastern Europe/Central Asia, Latin America, and Western Europe.^15,16,19-22^

Existing ADAPT survey questions were reviewed by a nine-member expert panel composed of multidisciplinary physicians representing pediatric oncology, PC, and public health from AP. The panel contextually and culturally adapted survey items to reflect PC provision in the region, resulting in the addition of nine questions to the existing survey. The final ADAPT-AP survey consisted of 74 items (69 quantitative, five qualitative; Data Supplement, Fig S1). Quantitative questions were assessed using a five-point Likert scale, with answers ranging from one (strongly disagree) to five (strongly agree). Alignment with WHO guidelines was assessed by asking respondents to answer multiple choice questions, with potential responses including never, rarely, sometimes, often, and always (Data Supplement, Table S1). Qualitative free-text prompts encouraged respondents to describe their experiences with PC provision.

Per the request of participating country teams, survey questions were translated into Indonesian, Japanese, Korean, Lao, Tetum, and Vietnamese (Data Supplement, Table S2). Translations were reviewed and pilot tested by the respective country team to ensure proper syntax, comprehension, and cultural relevance, with adjustments made on the basis of their feedback. Research teams from Indonesia, Japan, Laos, Republic of Korea, Thailand, Timor-Leste, and Vietnam anticipated respondents would prefer the option to respond to qualitative questions in their native languages. All other country teams opted to distribute and complete the survey in English.

### Survey Distribution Strategy

Routine meetings with each country team were scheduled to explore local pediatric PC provision and develop a tailored strategy for survey engagement and distribution, considerate of local context and care delivery. The survey was distributed in seven languages (Indonesian, English, Japanese, Korean, Lao, Tetum, and Vietnamese) to eligible physicians in 18 participating countries. Eligible physicians included those who routinely cared for children diagnosed with cancer, regardless of specialty. Each country team compiled a list of local physicians meeting this criterion for survey participation. Anonymized survey links were sent to eligible participants using Qualtrics.^25^

The surveys were launched in participating countries asynchronously in cohorts between February 2022 and February 2024 and remained open to data collection in each country for 4 months. Within the data collection period, country teams received updates on the number of completed surveys every 1-2 weeks; local teams then encouraged study participation (eg, reminders via e-mail or instant messaging services). Challenges related to instrument adaptation and translation, local study approval, and study distribution and implementation across AP were previously published.^26^

### Data Analysis

Descriptive statistics were used to report country-specific demographic data. Alignment with WHO guidance was assessed through 15 survey statements (ie, agreement or disagreement) and a percentage of alignment score was calculated for each respondent. The mean percentage of alignment of an individual physician's responses across the cohort was the primary outcome measured. To evaluate whether demographic factors were significantly associated with WHO alignment scores, univariable (simple linear regression analysis) and multivariable (multiple linear regression analysis) models were used. Univariable analyses included all demographic factors. Identified significant results were included in the multivariable analysis. Participating countries were grouped by World Bank income status.^27^ Responses to questions on five-point Likert scales were condensed into three categories (always or often, sometimes, and rarely or never) for secondary analysis to compare associations with demographic variables using the Pearson chi-squared or Fisher exact test. A two-tailed *P* value of <.05 was considered statistically significant. All summaries and analyses were conducted using SAS version 9.4 (SAS Institute).^28^

To analyze written free-text responses, items were translated to English by members of the research team (M.S., A.C) using Google Translate, when necessary.^29^ Native speakers from respective country teams reviewed and verified translations to ensure accuracy, consistency, and correct interpretation when concerns about translation quality arose.^29^ The codebook was developed using a priori (deductive) codes used in prior ADAPT studies and novel (inductive) codes from iterative review of participant responses to the following questions: What does PC mean to you? How is PC described to patients/families in your setting? (Data Supplement, Table S3).^8,12^ Each free-text response served as the unit of analysis. Codes were applied across transcripts by two members of the research team (M.S., A.C.) using MAXQDA software (WERBI, Berlin, Germany).^30^ Discrepancies were resolved by consensus with a third-party adjudicator (C.L.) when necessary.

## RESULTS

### Respondent Demographics

The survey was completed by 621 physicians from 18 countries across AP. The overall response rate was 27% (621/2,306), and the median country response rate was 30% (range, 11%-85% per country; Data Supplement, Table S2). Most respondents were >35 years of age (n = 484; 78%), identified as female (n = 373; 60%), and had >11 years of clinical experience following medical school graduation (n = 432; 70%). Almost half of respondents practiced in pediatric hematology/oncology (n = 265; 43%) and a quarter in general pediatrics (n = 156; 25%). More than 80% of respondents practiced at either a general hospital (n = 312; 50%) and or children's hospital (n = 205; 33%). Few respondents had received PC training (n = 220; 35%, range, 11%-82% by country) but did have access to general PC consultation (n = 452; 73%, range, 0%-100% by country) or a pediatric PC expert (n = 352; 57%, range, 0%-100% by country; Data Supplement, Table S5). Respondents with access to PC consultation primarily reported access to a physician (n = 340; 97%), nurse (n = 200; 57%), social worker (n = 135; 38%), or psychologist (n = 106; 30%) over other professions (Data Supplement, Table S4). Among respondents who had prior PC training (n = 220; 35%), most had completed a postgraduate (n = 103; 47%) or certificate course (n = 109; 50%; Data Supplement, Table S6). Almost all respondents had cared for a dying child during the previous year (n = 556; 90%). Respondent demographic characteristics are summarized in Table 1.

**TABLE 1:** Demographic Characteristics of Respondents Participating in the Assessing Doctors' Attitudes on Palliative Treatment Survey in Asia Pacific

Demographic Characteristic	Respondents, No. (%)
Setting-level characteristics	
Country	
Bhutan	18 (2.9)
Cambodia	10 (1.6)
India	103 (16.6)
Indonesia	33 (5.3)
Japan	50 (8.0)
Laos	42 (6.8)
Malaysia	49 (7.9)
Maldives	4 (0.7)
Myanmar	46 (7.4)
Nepal	25 (4.0)
New Zealand	18 (2.9)
Philippines	56 (9.0)
Republic of Korea	12 (1.9)
Singapore	44 (7.1)
Sri Lanka	11 (1.8)
Thailand	25 (4.0)
Timor-Leste	6 (1.0)
Vietnam	69 (11.1)
Primary institution	
General hospital	312 (50.2)
Children's hospital	205 (33.0)
Cancer hospital	79 (12.7)
Other	25 (4.1)
Access to PC consultation	
Yes	452 (72.8)
No	169 (27.2)
Access to pediatric PC expert	
Yes	352 (56.7)
No	269 (43.3)
Individual-(physician) level characteristics	
Age	
<35 years	137 (22.1)
≥35 years	484 (77.9)
Sex	
Female	373 (60.1)
Male	246 (39.6)
Prefer not to disclose	2 (0.3)
Primary medical specialty	
General pediatrics	156 (25.1)
Pediatric hematology/oncology	265 (42.7)
Pediatric PC	17 (2.7)
Other^a^	183 (29.5)
Years of experience	
0-10 years	189 (30.4)
≥11 years	432 (69.5)
Trained in PC	
Yes	220 (35.4)
No	401 (64.6)
Patients who died during care in previous year, No.	
0	65 (10.5)
1-5	322 (51.8)
≥6	234 (37.7)

Abbreviation: PC, palliative care.

a Other “primary medical specialties” includes: pediatric or adult anesthesiology; pediatric or adult surgery; adult or general PC; adult hematology and/or oncology; general internal medicine/family medicine; pediatric intensive care; pediatric ophthalmology; pediatric pulmonology; pediatric infectious diseases; adult, pediatric, or general radiation oncology; genetics; neurosurgery; ocular oncology; pediatric and/or adult orthopedic oncology.

### Components of PC

Nearly all respondents identified the role of PC to include reducing pain and suffering (n = 608; 98%) and providing psychological support (n = 591; 95%). Most respondents identified spiritual support, clarifying goals of care, assisting with decision-making or care transitions (ie, from hospital to hospice), and supporting bidirectional communication between the family and health care team as components of PC provision (Data Supplement, Table S7).

In response to the question, what does PC mean to you, respondents highlighted themes related to the positive attributes and components of PC, as well as ideal timing of PC integration in the care of a child diagnosed with cancer. Many described PC as a necessary service, which should be made accessible to all (physician, Philippines). Respondents associated PC with supporting patient dignity, offering compassion and love, and a celebration of life. Respondents also emphasized components such as multidisciplinary teamwork, end-of-life care, life prolongation, optimizing quality of life, and accompaniment. As one respondent shared, “PC means to be able to walk alongside a child and family and assess and provide treatment recommendations (physical, psychosocial, spiritual) for their own unique needs with the context/community that they find themselves in” (Physician, New Zealand). Although most respondents identified terminal illness or end-of-life care as appropriate timing of PC integration, some respondent quotes described the importance of PC throughout the childhood cancer continuum (ie, at diagnosis, disease progression). Quotes illustrating these themes are presented in the Data Supplement (Table S8).

Respondents were also asked, how is PC described to patients and families in your setting? Themes emerged related to how PC is introduced to families and the description of available PC services. Respondents described how PC was introduced variably by a child's primary medical team or deferred to PC consultants (eg, because of time constraints). Services were described broadly as a philosophy of care or more specifically as care focused on optimizing quality of life, aligning goals of care, or facilitating end-of-life care. A subset of respondents noted challenges in describing PC, including stigma—such as equating PC with abandoning treatment efforts. Quotes illustrating these themes are presented in the Data Supplement (Table S9).

### Alignment With WHO Guidance

Overall, respondents demonstrated a mean percentage alignment of 76% with WHO guidance on PC integration for children with serious illnesses (individual country scores ranging 48%-92%; Table 2 and Data Supplement, Table S2). Univariable analysis demonstrated that most demographic factors including respondent age, religiosity, spirituality, primary medical specialty, years of experience, prior PC training, and practice setting (ie, country income level, institution type, access to PC consultation) were significantly associated with respondent alignment to WHO guidance. Individual-level characteristics such as religiosity, spirituality, and prior PC training remained statistically significant in multivariable analysis. Setting-level characteristics (ie, country income level, institution type, or access to PC consultation) were not statistically significant in multivariable analysis.

**TABLE 2:** Association Between Respondent Demographic Factors and Response Alignment With WHO Guidance for Pediatric Palliative Care in Asia Pacific

Demographic Factor	Mean WHO Alignment, % (95% CI)	*P*	
		Univariate Model	Multivariate Model
Setting-level characteristics			
Country's income level			
Lower middle	75.3 (73.6 to 77.2)	<.0001	NS
Upper middle	77.4 (74.1 to 80.8)		
High	83.7 (80.5 to 86.9)		
Primary institution			
General hospital	75.8 (73.8 to 77.8)	.0452	NS
Children's hospital	77.9 (75.4 to 80.4)		
Cancer hospital	82.1 (78.1 to 86.1)		
Other	78.7 (71.5 to 85.8)		
Access to PC consultation			
Yes	79.0 (77.2 to 81.0)	.0086	NS
No	75.2 (73.0 to 77.4)		
Individual (physician)-level characteristics			
Age			
<35 years	69.6 (66.6 to 72.6)	<.0001	NS
≥35 years	79.6 (78.0 to 81.2)		
Sex			
Female	77.0 (75.1 to 78.8)	.1216	NA
Male	78.2 (76.0 to 80.5)		
Prefer not to disclose	53.3 (28.0 to 78.6)		
Religious			
Yes	78.0 (76.3 to 79.8)	<.0001	.0448
Neutral	70.7 (66.7 to 74.7)		
No	80.3 (77.1 to 83.4)		
Prefer not to disclose	53.3 (35.7 to 71.0)		
Spiritual			
Yes	79.7 (77.9 to 81.4)	<.0001	.0328
Neutral	70.6 (67.3 to 73.8)		
No	76.2 (72.7 to 79.7)		
Prefer not to disclose	73.3 (53.0 to 93.7)		
Primary medical specialty			
General pediatrics	70.0 (67.2 to 72.8)	<.0001	NS
PHO	79.1 (76.9 to 81.2)		
Pediatric PC	92.9 (84.5 to 100.0)		
Other^a^	79.8 (77.3 to 82.4)		
Years of experience			
0-10 years	71.2 (68.7 to 73.8)	<.0001	NS
≥11 years	80.1 (78.4 to 81.8)		
Trained in PC			
Yes	81.8 (79.4 to 84.1)	<.0001	.0007
No	75.0 (73.2 to 76.8)		
Patients who died during care in previous year			
0-5	76.5 (74.6 to 78.3)	.0988	NA
≥6	79.0 (76.6 to 81.3)		

Abbreviations: NA, not applicable; NS, not significant; PC, palliative care.

a Other “primary medical specialties” includes: Pediatric or adult anesthesiology; pediatric or adult surgery; adult or general PC; adult hematology and/or oncology; general internal medicine/family medicine; pediatric intensive care; pediatric ophthalmology; pediatric pulmonology; pediatric infectious diseases; adult, pediatric, or general radiation oncology; genetics; neurosurgery; ocular oncology; pediatric and/or adult orthopedic oncology.

More than 90% of respondents correctly identified that PC involvement results in greater focus on quality of life and symptom management and that early PC integration improves interdisciplinary communication (Data Supplement, Table S10). Common misconceptions and misalignments with WHO guidance included difficulty determining when a patient and family would benefit from PC consultation (54% misaligned), belief that early PC consultation increases parental burden and anxiety (40% misaligned), and misperceptions that PC is synonymous with end-of-life care (35% misaligned; Data Supplement, Table S10).

### Physician Confidence in PC Provision

**FIG 1:**
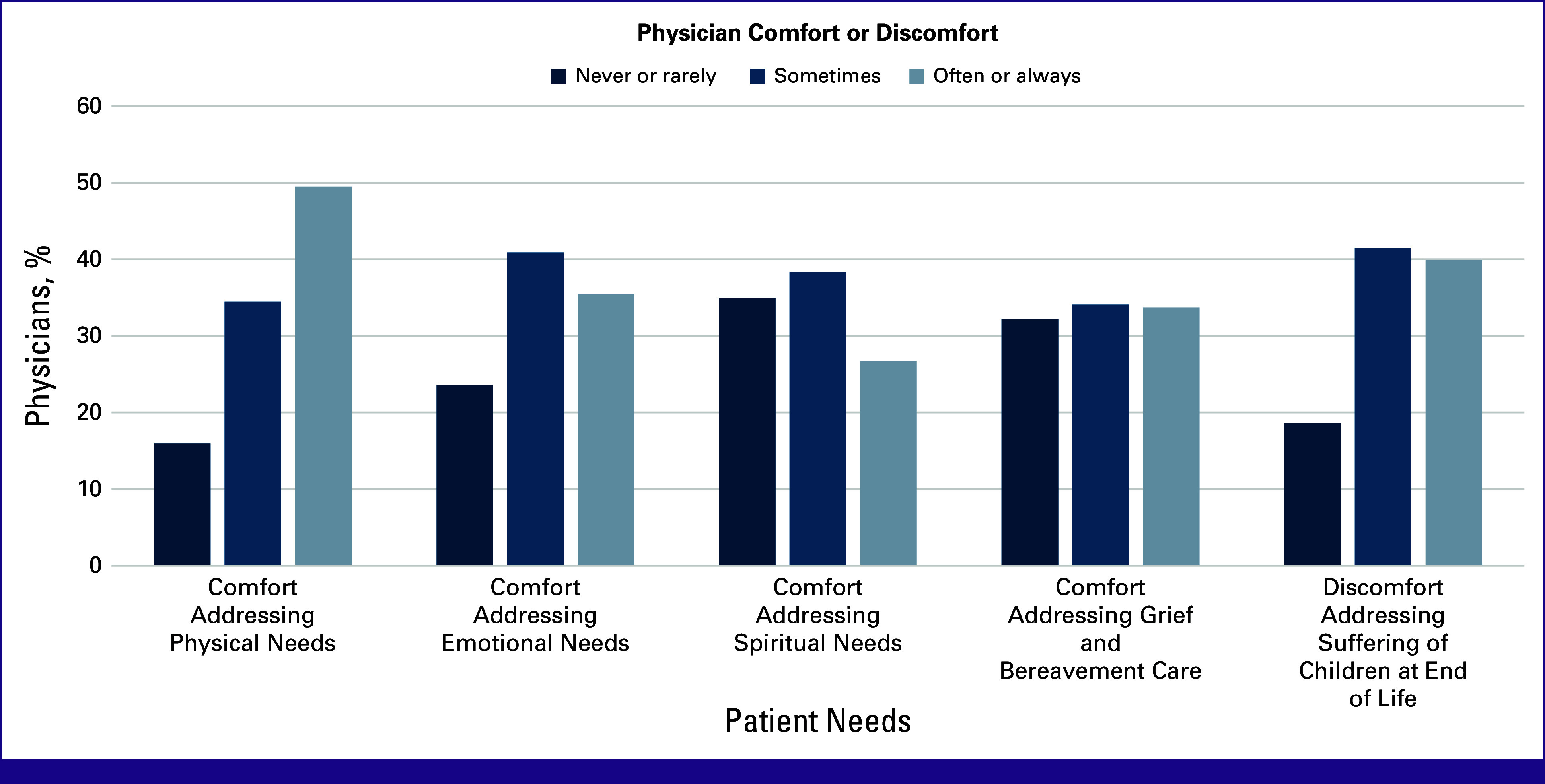
Physician comfort in addressing palliative care needs of children diagnosed with cancer and their families. Participant responded to survey items using a five-point Likert scale that was collapsed into three categories for presentation.

Most respondents described discomfort with addressing PC needs of children and their families (Fig 1). Among respondents, only 50% (n = 308), 36% (n = 221), 27% (n = 166), and 34% (n = 209) expressed feeling often or always comfortable addressing physical, emotional, spiritual, and grief/bereavement needs of patients and families, respectively. Notably, more than 20% of respondents never or rarely felt comfort addressing emotional (n = 146; 24%), spiritual (n = 217; 35%), or grief and bereavement needs (n = 200; 32%). Additionally, 40% of respondents (n = 248) often or always felt burdened by their inability to control the suffering of children at end of life.

Prior PC training increased respondent confidence in assessing and addressing the needs of patients and families (Table 3). Respondents with prior PC training (n = 220; 35%) felt more comfortable addressing physical (n = 133 [61%] *v* n = 175 [44%]; *P* < .01), emotional (n = 108 [49%] *v* n = 113 [28%]; *P* < .01), spiritual (n = 79 [36%] *v* n = 87 [22%]; *P* < .01), and grief and bereavement (n = 103 [47%] *v* n = 106 [26%]; *P* < .01) needs. Respondents with prior PC training felt less burdened by their inability to control suffering of children at end of life (n = 51 [23%] *v* n = 65 [16%]; *P* < .01).

**TABLE 3:** Physician Confidence in Assessing Palliative Care Needs of Children Diagnosed With Cancer and Their Families, Stratified by Receipt of Prior Palliative Care Training

Survey Item	Previous Palliative Care Training, No. (%)		*P*
	Yes (n = 220)	No (n = 402)	
I feel confident assessing and treating the physical needs of pediatric patients with serious incurable illness			
Never or rarely	26 (11.8)	73 (18.2)	.0003
Sometimes	61 (27.7)	153 (38.2)	
Often or always	133 (60.5)	175 (43.6)	
I feel confident assessing and treating the emotional needs of pediatric patients with serious incurable illness and their families			
Never or rarely	41 (18.6)	105 (26.2)	<.0001
Sometimes	71 (32.3)	183 (45.6)	
Often or always	108 (49.1)	113 (28.2)	
I feel confident taking care of the spiritual needs of pediatric patients and their families with serious incurable illness			
Never or rarely	60 (27.3)	157 (39.2)	.0002
Sometimes	81 (36.8)	157 (39.2)	
Often or always	79 (35.9)	87 (21.7)	
I feel confident providing grief and bereavement care to the families of children who die			
Never or rarely	58 (26.4)	142 (35.4)	<.0001
Sometimes	59 (26.8)	153 (38.2)	
Often or always	103 (46.8)	106 (26.4)	
I have felt burdened by my inability to control the suffering of children at the end of life			
Never or rarely	51 (23.2)	65 (16.2)	.0085
Sometimes	98 (44.5)	160 (39.7)	
Often or always	71 (32.3)	177 (44.1)	

### Physician Practices and Attitudes Toward PC Provision

More than half (n = 365; 59%) of respondents indicated that PC was available when needed for children diagnosed with cancer in their settings. However, 68% (n = 423) reported that quality of life was often overlooked in favor of cancer-directed treatment, and 38% (n = 236) believed that PC consultation occurred too late in a child's treatment course. Almost one third of respondents (29%; n = 180) shared that physicians in their settings typically continued to recommend curative treatment to children with incurable cancer, even when the therapy was ineffective or unlikely to prolong a child's life. Almost all respondents emphasized that a greater focus on PC in medical education was necessary to improve PC access (n = 584; 94%) and desired more PC education (n = 570; 92%; Data Supplement, Table S11).

## DISCUSSION

This mixed-methods study reports physician perceptions toward PC integration into childhood cancer care across 18 countries in AP. Most respondents correctly described and identified core components of PC provision, although some maintained PC-related misconceptions (ie, PC was indicated only for children at end of life) and difficulty knowing when to consult local PC teams. Moreover, many respondents reported discomfort with addressing PC needs of patients and their families (ie, emotional, spiritual, grief/bereavement) and feeling burdened in their inability to control suffering of children at end of life. Respondents with prior PC training demonstrated improved skills, confidence, and resiliency (ie, WHO alignment, confidence addressing PC needs, provider burden). Almost all expressed the importance and desire for further PC education.

The findings of this study must be considered in the context of findings from ADAPT studies conducted in other regions.^15,16,19-22^ Median WHO alignment in AP was similar to Eastern Europe/Central Asia (70%) but less than that reported in Latin America (83%) and Western Europe (83%). Notably, physician comfort in AP to address physical (50%), emotional (36%), spiritual (27%), and grief/bereavement needs (34%) were similar or lower than respondents in Eastern Europe/Central Asia, Latin America, and Western Europe, which reported the following ranges of physician comfort for addressing physical (50%-84%), emotional (34%-64%), spiritual (29%-42%), and grief/bereavement (25%-49%) needs. Regional differences are also noted regarding burden addressing suffering at end of life, reported by 40%, 60%, 32%, and 25% of respondents in AP, Eastern Europe/Central Asia, Latin America, and Western Europe, respectively. This variance underscores the importance of region-specific interventions to strengthen physician understanding and application of core PC competencies. Analysis is ongoing to characterize and describe challenges faced providing PC in AP, and efforts are underway to expand the ADAPT study and compare findings across all world regions.

Importantly, >90% of ADAPT respondents reported desire for further education and training in PC, regardless of region.^15,19,21,22^ ADAPT-informed educational initiatives in Eastern Europe/Central Asia effectively improved physician confidence to address PC needs of children and their families.^18^ In this study, findings reported significant increases in confidence by respondents with prior PC training when assessing and addressing needs of patients and families compared with those without PC training. Moreover, physicians with prior PC training reported feeling less burdened when unable to control patient suffering at end of life, suggesting that PC training enhances resiliency and mitigates risk of undesired provider outcomes (eg, burnout). Collectively, these findings demonstrate the importance of regional and country-level PC education initiatives. Our study findings offer an opportunity to inform future initiatives to teach clinicians' both primary and secondary (specialist) PC skills and competencies, leveraging existing regional collaborations.^31-39^ Future research is needed to assess the impact of PC education on healthcare delivery and patient- and family-centered outcomes in AP.^14,40-42^

This study highlights the urgent need for targeted, region-specific strategies to strengthen PC capacity and advocacy across AP. Although 73% of respondents reported access to multidisciplinary PC services, availability varied widely—from no access in Timor to all respondents reporting access in the Republic of Korea, Singapore, and Thailand. Addressing these gaps requires understanding the PC needs of children with cancer and their families and developing services and professional development opportunities tailored to those needs.^18,42,43^ Advocacy should focus on reducing stigma, clarifying the role of PC throughout the disease trajectory, and promoting its integration into pediatric cancer care, including incorporation into national pediatric health care policies.^3-11^ These findings will inform country- and region-level reports that include clinical and policy recommendations. Similar reports generated from ADAPT studies conducted in Eastern Europe/Central Asia and Latin America have successfully used as evidence-based advocacy tools that the bridge the gap between research, policy, and health care delivery.^17,44^ Continued collaboration is essential to build community, share experiences, and improve equitable access to PC across the region.

This study had several limitations. As leaders in their respective countries, country teams were asked to identify eligible physicians for study participation. Strategies varied country to country, based on expert knowledge of local health care structures. Although approaches were comprehensive, it is possible that a subset of physicians who routinely care for children with cancer were not invited to participate. Although 621 physicians completed the survey, the overall response rate was low (27%; median 30%, country range, 11%-85%). Countries with diverse workforces were included, ranging from small (Timor Leste—22 invited physicians) to large sizes (India—739 invited physicians; Data Supplement, Table S2). As a result, recruiting physicians proved difficult despite regular reminders and a 4-month participation window.^26^ Survey participation may also have been affected by survey fatigue and lack of protected time for research in the setting of high clinical burdens. Additionally, this study included physicians, who comprise only one group of childhood cancer providers, and future work will assess multidisciplinary clinician, patient, and caregiver perspectives.

In summary, physicians caring for children with cancer in AP have a significant unaddressed need and desire for PC training. Although physicians understood core PC components, many expressed discomfort providing PC to patients and families and felt burdened by their inability to control patient suffering. Physicians with prior PC training reported greater confidence in PC delivery and enhanced resiliency. Understanding the perspectives of clinicians who provide PC is crucial as health care systems and local teams consider expanding PC programs. These findings will inform future regional and country-level education, advocacy, capacity-building, and research initiatives to enhance PC access. Ultimately, more equitable access to PC across AP has the potential to improve patient and family outcomes and clinician confidence in providing high-quality childhood cancer care across the illness course.
